# Study of factors affecting the blend of virgin and aged asphalt binder based on molecular dynamics simulation

**DOI:** 10.1371/journal.pone.0328441

**Published:** 2025-08-12

**Authors:** Fuming Liu, Aoyun Qi

**Affiliations:** 1 College of Civil and Architectural Engineering, Nanchang Institute of Technology, Nanchang, Jiangxi, China; 2 College of Civil Engineering, Chongqing Jiaotong University, Chongqing, China; Oregon State University, UNITED STATES OF AMERICA

## Abstract

To understand the blending behavior of virgin and aged asphalt binder in thermally recycled asphalt mixtures, a series of studies on the blending behavior of virgin and aged asphalt based on molecular dynamics simulations have been performed. A blend model was developed for virgin asphalt binder and aged asphalt binder with rejuvenator system, which is saturated with 12 different molecules. In-depth analysis of microscopic blending mechanisms through the effects of rejuvenator content, simulation time, temperature and aged asphalt binder content on the degree of blending of virgin and aged asphalt binder. Using Materials Studio, the blend of virgin and aged asphalt binder under different conditions was quantitatively analyzed. It was found that the degree of blending of virgin and aged asphalt increased gradually with the content of rejuvenator, but the degree of blending decreased slightly when increased to 15%. The degree of blending gradually increased with the increase of time and also with the increase of temperature during the simulation time of 2000 ps. In terms of aged asphalt content, the degree of blending of virgin and aged asphalt gradually decreased with the increase of aged asphalt content. And through the fitting analysis found that the order of correlation between the four factors and the degree of blending was: simulation time > aged asphalt content > temperature > rejuvenator content. To verify the reliability of the results of the molecular dynamics simulation, the performance of recycled asphalt was verified using the asphalt rheological properties test (DSR) and the three major indexes (needle penetration, softening point, and ductility) tests. The results of the combined rheological property test and the three major index tests were found to be consistent with the order of influence of the four factors obtained in the molecular dynamics simulation. Therefore, it can be concluded that the use of molecular dynamics simulation in this study to analyze the blend behavior of virgin and aged asphalt in recycled asphalt is relatively accurate.

## 0 Introduction

Road construction is an important foundation for urban infrastructure development. In order to cope with the increasingly severe sustainability challenges, many renewable materials have been gradually applied to highway engineering and Reclaimed Asphalt Pavement (RAP) has received considerable attention in road construction [1,2].The application of RAP is able to reduce the consumption of non-renewable resources, which is in line with the concept of recycling of large solid wastes for comprehensive utilization to implement economy proposed in the outline of the current ‘14th Five-Year Plan’ [3]. However, compared with virgin asphalt binder, the asphalt binder in RAP ages during service and becomes hard and brittle, losing its binding effect on the aggregate [4]. At present, the development of plant-mixed thermal recycling technology faces two difficulties: one is the low utilization rate of RAP, which restricts the development of the technology; the second is that the blending mechanism of virgin and aged asphalt at the microscopic level has not yet been clarified, which affects the improvement of the performance evaluation system of recycled asphalt blends. Therefore, it is necessary to deeply analyze the microscopic blending mechanism of recycled asphalt materials from the microscopic level, which can provide theoretical support for the optimization of the recycling process.

To minimize the negative effects of asphalt binder ageing, during the hot rejuvenation of asphalt pavements, a certain content of virgin asphalt binder and rejuvenator is usually added depending on the degree of ageing [5]. Since the performance of recycled asphalt pavements with rejuvenator is like that of virgin asphalt binder, RAP has potential economic and environmental value. Wang et al. [6] showed that the recycled asphalt binder obtained by adding rejuvenator has similar low-temperature fracture performance as that of matrix asphalt binder, and its fatigue performance is even better than that of matrix asphalt binder. A rejuvenator is a chemical or biological-based substance that can bring the ratio of components in aged asphalt binder back to equilibrium [7].Sun et al [8] analyzed the effect of diffusion coefficient of aged asphalt binder on its rejuvenating effect from the point of the molecular structure. Bio-based rejuvenator is a kind of asphalt rejuvenator developed using biological resources, and the addition of bio-rejuvenator extracted from waste cooking oil resulted in better ductility of bio- rejuvenated asphalt binder than conventional asphalt binder at low temperatures [9].

There is still uncertainty about the state of blending of virgin and aged asphalt binder in hot recycled asphalt binder at the microscopic level. Most scholars hold the following three conclusions about the degree of blending of virgin and aged asphalt binder [10,11]: black aggregate, partial blending and complete blending. The incomplete blending of virgin and aged asphalt binder has an adverse effect on the performance of recycled asphalt binder, which leads to the deterioration of the performance of recycled asphalt mixtures. China’s current asphalt pavement rejuvenation technical specifications [12] on the hot recycled asphalt mixture are mainly considered less than 30% of the RAP content, and the pavement structural design is based on the concept of complete blending of virgin and aged asphalt binder. However, according to theoretical research and actual construction process [13] found that only part of the aged asphalt binder in recycled asphalt mixtures blend with the virgin asphalt binder, which will lead to insufficient performance of recycled asphalt, limiting the increase of RAP content. So many scholars are committed to the study of the degree of blending between virgin asphalt binder, aged asphalt binder, and rejuvenator at multiple scales. Navaro [14] investigated the degree of blending behavior of virgin aged and asphalt by NT-FTIR coupling technique, and the results showed that the degree of blending had a significant correlation with the mixing temperature and mixing time, and proposed the concept of DOB for the degree of blending. Bowers [15] investigated different levels of extracted asphalt by a layered extraction test combined with the GPC and FTIR techniques and found that the three-layer asphalt system showed the characteristic of partial blending, and the complex modulus and viscosity values of asphalt showed a decreasing trend from the inner layer to the outer layer. In terms of asphalt rheological properties, Mohammad Zia Alavi [16] assessed the degree of blend and interaction between virgin and aged asphalt using rheological and fracture measurements. In addition, an analysis of the composite shear modulus revealed that the mechanical modulus and high temperature properties of recycled asphalt increased in direct proportion to the percentage of aged asphalt. Sunny Lewis [17] selected three different RAP blends, 0%, 50% and 100%, and evaluated their effects on the properties of the asphalt mixture and binder by means of semicircular bending tests and rheological tests, respectively. The amount of RAP binder in the final blend was found to have a significant effect on crack resistance.

For quantitative analysis of the degree of blending of virgin and aged asphalt binder, Prashant Shirodkar [18] and others proposed to use the ‘degree of blending’ to express the proportion of aged asphalt binder blend with virgin asphalt binder to the total amount of aged asphalt binder; Norton [19] et al. defined the proportion of aged asphalt binder that acts as a binder in an asphalt mix as ‘degree of blending’; Gottumukkala [20] et al. defined ‘degree of blending’ as the ratio of the weight of the aged asphalt binder blended with the virgin asphalt binder to the total weight of the aged asphalt binder; Ding [21] et al. defined the proportion of aged asphalt binder that flakes from the surface of the old aggregate and blended well with the rejuvenator as ‘RAP binder mobilization rate’.

In molecular dynamics simulations, Ding HeYang [22] used molecular dynamics software to establish the diffusion models of two types of biological rejuvenator, straight-chain and aromatic, with asphalt binder, and found that the addition of biological rejuvenator was able to reduce the degree of agglomeration of the four-component of asphalt binder. Zhu Yajing [23] et al. simulated the blending behavior of rejuvenator with virgin and aged asphalt binder by molecular dynamics simulation, and found that there were differences in the diffusion of virgin and aged asphalt binder with rejuvenator at different depths of the recycled asphalt binder surface layer, and it was difficult to eliminate them in the short term. Molecular dynamics simulation can analyze the miscibility behavior of virgin and aged asphalt at the atomic and molecular levels, helping to understand their interactions and structural changes. Molecular dynamics simulation can reduce the number of experimental tests, thereby saving time and costs.

In summary, the degree of blending of virgin and aged asphalt binder can be analyzed by a variety of methods, but all have certain shortcomings and are not yet clearly understood. To ensure the performance of recycled asphalt binder, it is necessary to quantitatively analyze from the molecular level to determine the degree of blending between virgin and aged asphalt binder. This study is based on the four-component twelve-molecular model of asphalt binder, to establish the blending model of the virgin and aged asphalt binder and rejuvenator system, and to investigate the effects of rejuvenator content, simulation time, temperature and aged asphalt binder content on the degree of blending of the virgin and aged asphalt binder in the hot recycled asphalt binder.

## 1 Molecular dynamics simulation

Molecular dynamics simulation (MD) is a method of defining intermolecular interactions to obtain dynamic information in the process of molecular motion, which can predict the macroscopic physical properties of asphalt binder from the molecular level and provide a theoretical basis for the study of the physicochemical properties of asphalt binder. Using molecular dynamics to simulate the blending process of virgin and aged asphalt binder, the coordinates and momentum of particles at different moments can be obtained, and the blending mechanism of virgin and aged asphalt binder can be analyzed from the microscopic level. Its main steps include: initial model construction, force field parameter setting, model structure optimization, system relaxation, dynamic simulation, and performance calculation. In this study, Materials Studio software is used for simulation and calculation.

### 1.1 Simulation parameters and input conditions

The Condensed-phase Optimized Molecular Potentials for Atomistic Simulation Studies II (COMPASS II) force field was chosen during the molecular simulations to accurately predict the material properties of various compounds in the isolated and condensed phases. The use of periodic boundary conditions in the simulations is equivalent to scaling up the original box to infinity, which is extremely close to the actual situation and eliminates the influence of boundary effects [24]. The control of temperature and pressure has a crucial influence on the system properties, and the commonly used conditional constraint systems are Microcanonical ensemble (NVE ensemble), Canonical ensemble (NVT ensemble), Isothermal–isobaric ensemble (NPT ensemble), Isenthalpic–isobaric ensemble (NPH ensemble), and Generalized ensembles. At the beginning of the simulation, the NVT ensemble was used first, so that the asphalt system quickly reaches the specified temperature under the condition of volume stability, the energy tends to stabilize. When the energy in the system gradually tends to stabilize, then the NPT ensemble is used to make the system under the condition of relatively constant temperature for its volume compression. The Nose-Hoover method was used to control the temperature, Andersen method to control the pressure, the time step was set to 1 fs, and the cutoff radius was 15.5 Å.

### 1.2 Molecular modelling of virgin and aged asphalt binder

**Fig 1:**
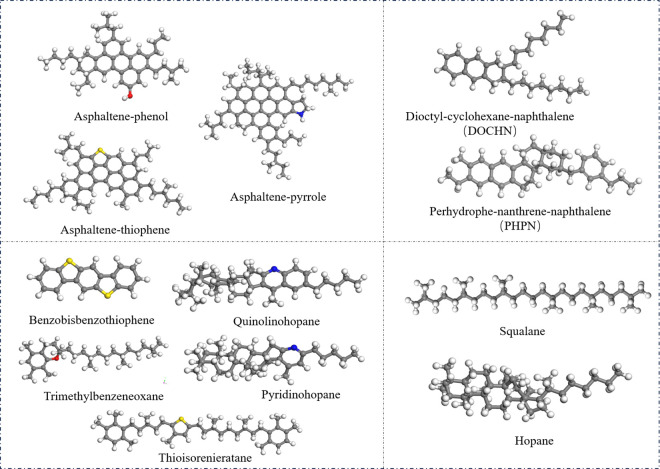
Molecular structure of virgin asphalt binder.

Li and Greenfield proposed AAA-1, AAK-1 and AAM-1 asphalt binder models in 2014, among which the density and thermodynamic properties of AAA-1 asphalt binder model are closer to the actual asphalt binder model. Therefore, a four-component model based on AAA-1 is used in this study [25], as shown in Fig 1. The four components in the figure correspond sequentially to the asphaltenes on the upper left, naphthene aromatic on the upper right, polar aromatic on the lower left, and saturates on the lower right, where grey denotes the carbon(C), white denotes the hydrogen(H), red denotes the oxygen(O), yellow denotes the sulfur (S), and blue denotes the nitrogen(N).

**Fig 2:**
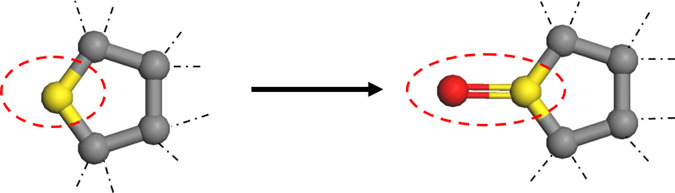
Sulfoxide functional group formation process in asphalt binder ageing.

**Fig 3:**
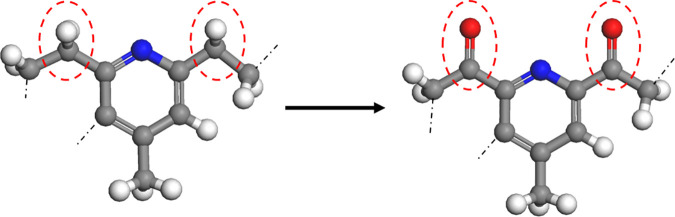
Ketone functional group formation process in asphalt binder ageing.

**Fig 4:**
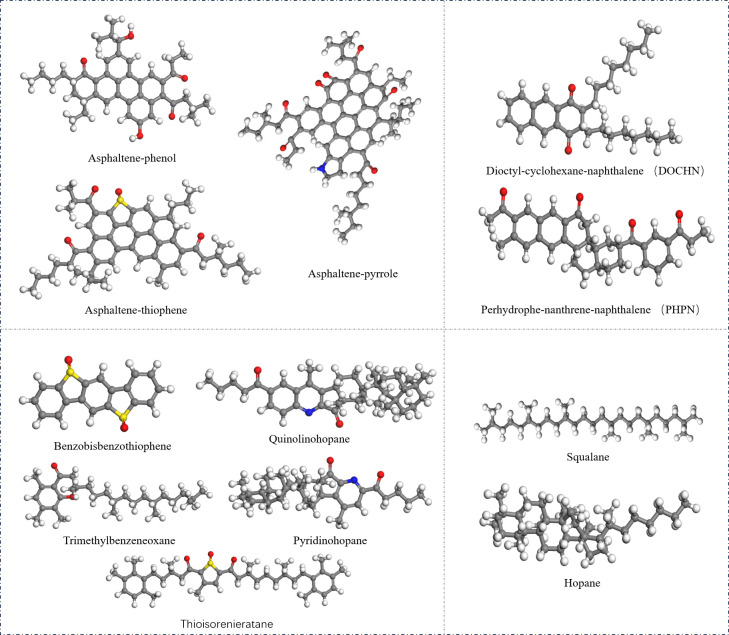
Molecular structure of aged asphalt binder.

Experimental studies have shown [26] that the saturates fraction is the least sensitive to oxidative ageing and, due to the lack of polar atoms, it rarely changes with the external environment and ageing time. Therefore, only asphaltenes, naphthene aromatic and polar aromatic fractions have sensitive functional groups that may undergo oxidative ageing. The oxidative aging reaction of asphalt binder is mainly the reaction between oxygen and the sensitive functional groups in asphalt binder to form carbonyl compounds. From a microscopic point of view, the aging process of asphalt binder is reflected by the changes of the elements and chemical bonds in the molecular structure. Researchers have found that oxidative aging of asphalt binder produces sulfinyl groups (derived from the reaction of sulfide with oxygen, shown in Fig 2 and ketyl groups [27] (derived from the substitution of oxygen atoms for hydrogen atoms on benzylic carbons, shown in Fig 3. The relative contents of the sulfoxide and ketone functional groups can be measured based on FTIR and used to determine the number of sulfoxide and ketone functional groups in aged asphalt binder [28]. In this study, we refer to the characterization method of aged asphalt binder proposed by Xu et al [26], and the molecular structure of aged asphalt binder is shown in Fig 4.

This study mainly analyzes the effect of different aged asphalt binder content, in order to accurately characterize the chemical and physical structure of asphalt binder, on the basis of the previous research results [29], different aged asphalt binder content is represented by adjusting the weight ratio of molecular components. The detailed components of the constructed molecular model of virgin and aged asphalt binder are listed in Table 1 and Table 2. After aging of asphalt binder, the ratio of the four components changed, in which the ratio of saturates and naphthene aromatic fractions decreased, and the ratio of asphaltenes and polar aromatic increased. From the four-component test, the four-component ratio of asphalt binder after aging is 18.6% for saturated fraction, 28.1% for naphthene aromatic fraction, 37.2% for polar aromatic fraction, and 16.1% for asphaltene fraction. This study chose 25%, 35%, 45% of the three aged asphalt binder content according to the aging asphalt binder four-component ratio of the aged asphalt binder 12 molecular structure of each content of molecular ratios were calculated, the results are shown in Table 2.

**Table 1:** Molecular structure of virgin asphalt binder.

Components	Molecular	Number	Component ratio (%)
saturates	C_30_H_62_	8	22.44
	C_35_H_62_	7	
naphthene aromatics	C_35_H_44_	14	41.83
	C_30_H_46_	15	
polar aromatics	C_40_H_59_N	1	24.31
	C_40_H_60_S	2	
	C_18_H_10_S_2_	13	
	C_36_H_57_N	2	
	C_29_H_50_O	2	
asphaltenes	C_42_H_54_O	2	11.42
	C_66_H_81_N	1	
	C_51_H_62_S	2	

**Table 2:** Molecular structure of aged asphalt binder.

Components	Molecular	Number of molecules		
		45%	35%	25%
saturates	C_30_H_62_	5	3	2
	C_35_H_62_	5	4	2
naphthene aromatics	C_35_H_36_O_4_	8	6	3
	C_30_H_42_O_2_	6	5	2
polar aromatics	C_40_H_55_NO_2_	2	1	1
	C_40_H_56_O_3_S	5	4	2
	C_18_H_10_O_2_S_2_	14	8	3
	C_36_H_53_NO_2_	2	1	1
	C_29_H_48_O_2_	2	1	1
asphaltenes	C_42_H_46_O_5_	2	1	1
	C_66_H_69_NO_7_	2	1	1
	C_51_H_54_O_5_S	2	2	1

**Fig 5:**
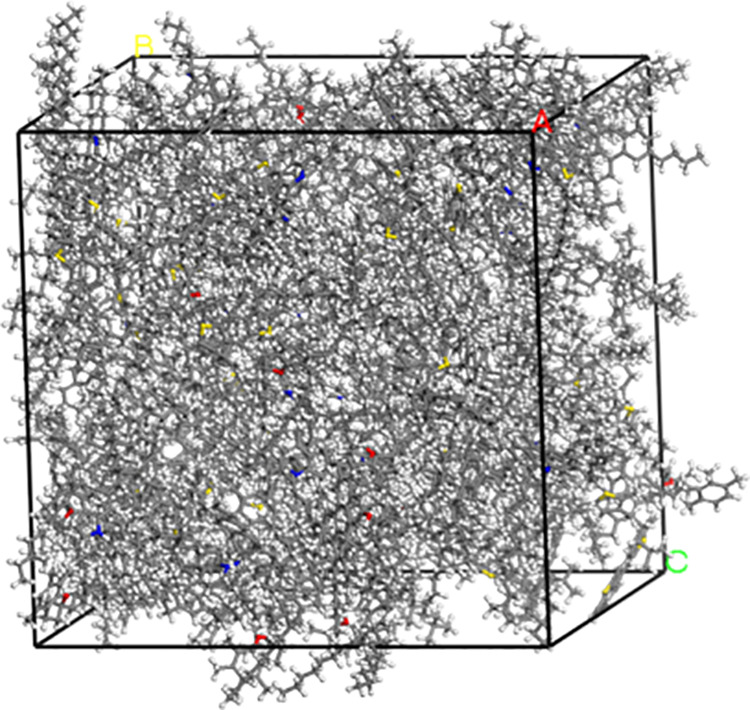
Amorphous cell model of virgin asphalt binder.

**Fig 6:**
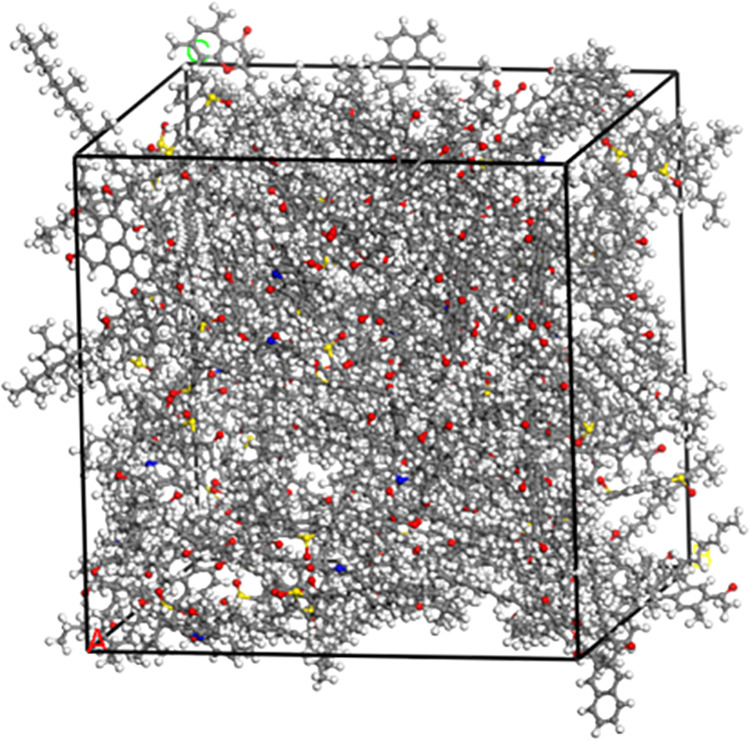
Amorphous cell model of aged asphalt binder.

Molecular models of the virgin and aged asphalt binders were constructed according to the number of molecules in Table 1 and Table 2, respectively. The Amorphous Cell module was used to construct the asphalt binder model with a density of 0.1 g/cm^3^ and the temperature was set at 298 K. The preliminary asphalt binder model was subjected to a 20,000-step geometry optimization to minimize the energy of the system and improve the stability. Following this, a simulation of 500 ps were simulated under the NVT ensemble to reach the specified temperature. After stabilization, 500 ps of dynamic simulation were performed on the asphalt binder model under the NPT ensemble with a pressure of 0.0001 GPa to compress the model volume, so as to construct the virgin and aged asphalt binder models that reached equilibrium, as shown in Figs 5 and 6. In molecular dynamics simulations, picoseconds (ps) are commonly used as a time unit to describe the time scale of molecular motion or reactions.

Fig 5 shows the amorphous cell model of the virgin asphalt binder after the asphalt model has reached equilibrium, with a side length of 38.04 Å, a total number of atoms in the model of 5271, and a total energy of the model of 12415.70 kcal/mol. Fig 6 shows the amorphous cell model of the aged asphalt binder with 35% content, for example, with a side length of 31.09 Å, a total number of atoms in the model of 2,755, and a total energy of the model of 7218.41 kcal/mol.

### 1.3 Molecular modelling of rejuvenator

Rejuvenator can both supplement the lightweight component and reduce the agglomeration phenomenon caused by oxidative aging in asphalt binder, Ding et al [30], Xu et al [31] investigated the rejuvenation effect of different rejuvenator on aged asphalt binder. Bio-rejuvenator is an asphalt additive developed from biological resources, and the addition of bio-oil significantly improves the fatigue resistance of asphalt mixtures and shows good durability in the service [22]. Bio-rejuvenator can be extracted from waste cooking oil and blended into asphalt binder it was found that bio-asphalt mixtures have higher ductility than conventional mixtures at lower temperatures [9].Fini et al [32] and Xiu et al [33] based on the analyzes of gas chromatography-mass spectrometry (GC-MS) and nuclear magnetic resonance (NMR) data concluded that bio-rejuvenator contains a large number of amide and nitrogenous functional groups, and Pahlavan et al [34,35] proposed seven molecular structures for the main components of the bio-rejuvenator.

**Fig 7:**
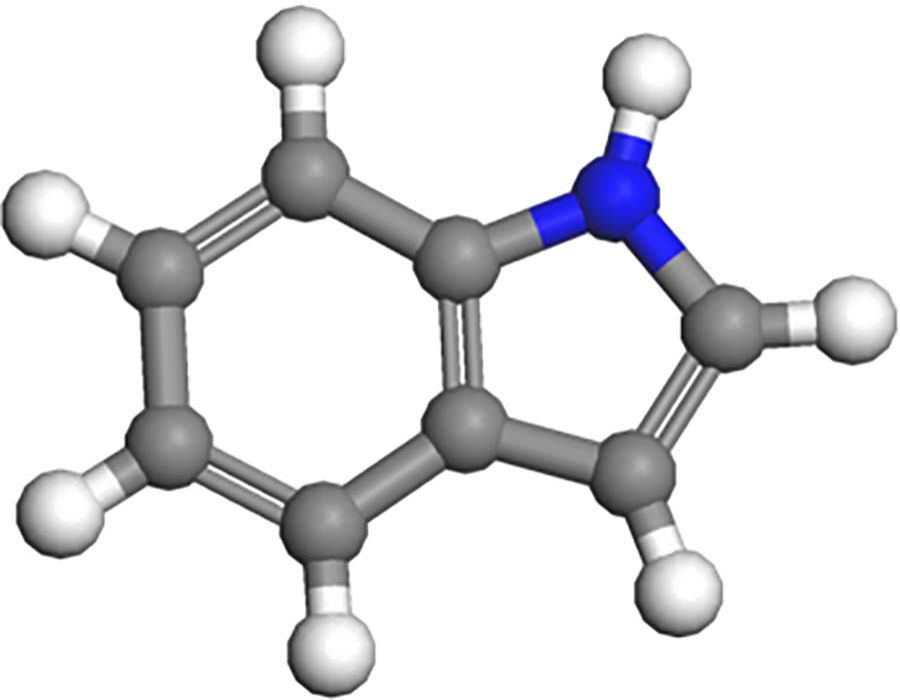
Molecular model of bio-oil rejuvenator 0f BR-5.

**Fig 8:**
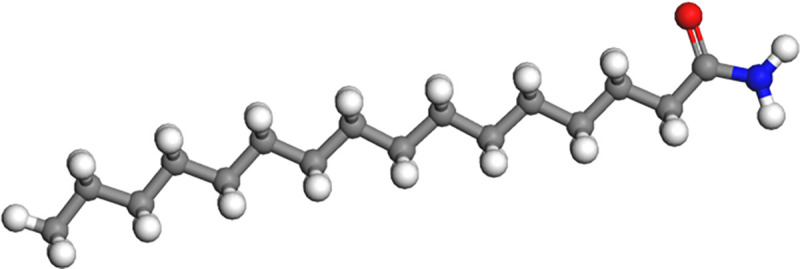
Molecular model of bio-oil rejuvenator of BR-1.

In order to make this study more comprehensive, taking into account the differences in the extraction sources and chemical structures of the rejuvenator, two typical molecular structures (BR-1: C_16_H_33_NO, BR-5: C_8_H_7_N) were selected from the seven molecular structures of the rejuvenator proposed by Pahlavan et al [34,35] (No.: BR-1 ~ BR-7), as shown in Figs 7 and 8. BR-1 has an amide functional group, a straight chain structure; BR-5 has a nitrogen-containing functional group, an aromatic compound structure, with the ratio of 1:1 of the two blended into the aged asphalt binder. This study in the modelling according to the hot rejuvenation construction process firstly the rejuvenator molecules are blended into the aged asphalt binder, rejuvenator blending amount selection of 5%, 10% and 15% of the three.

### 1.4 Model simulation reliability verification

#### 1.4.1 Verification of model density and solubility parameters.

**Fig 9:**
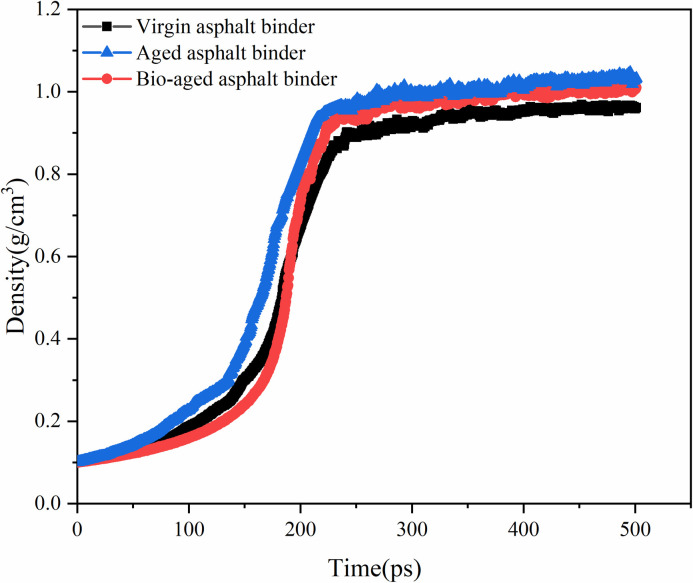
Density variation in the dynamics simulation.

Deriving the density data of the NPT system during the simulation process, it is found that the density of the model tends to stabilize after 300 ps, the density of the aged asphalt binder is stable at 1.031 g/cm3, the density of the virgin asphalt binder is stable at 0.975 g/cm3, and the density of the 15% rejuvenator-aged asphalt binder system is stable at 1.009 g/cm3, as shown in Fig 9. Comparison with the experimental values shows that the modelled density values are basically within a reasonable range, as shown in Table 3, and it can be assumed that the system has reached a relative equilibrium state.

The solubility parameter is an important parameter for measuring the compatibility of different substances and can quantitatively characterize the strength of interactions between substances, and its value is equal to the square root of the cohesive energy density The cohesive energy density is equal to the energy required to eliminate inter-molecular interactions per unit volume of the system, and the larger the cohesive energy density is, the stronger the intermolecular interactions are. The solubility parameter is calculated by Eq.(1), (2) [36]:


$$CED=\frac{E_{coh}}{V}$$ (1)

$$\delta =\sqrt{CED}$$ (2)

Where CED is the cohesive energy density of the material; E_coh_ is the total cohesive energy of the system; V is the total volume of the system; δ is the solubility parameter.

The solubility parameters of the virgin and aged asphalt binder were calculated using the Forcite module and compared with the experimental values as shown in Table 3.

**Table 3:** Comparison of calculated properties of asphalt binder materials with test values.

Index	Simulation value			Experimental value
	Virgin asphalt binder	Aged asphalt binder	Aged asphalt binder −15% rejuvenator	
density(g/cm^3^)	0.975	1.031	1.009	0.95 ~ 1.08 [26]
solubility parameter ((J/cm3)^1/2^)	17.96	19.16	18.55	13.3 ~ 22.5 [37]

From the thermodynamic point of the amorphous cell model to verify the nature of the simulation of the density and solubility parameters obtained with the previous experimental data, found that the results are close to the experimental data, indicating that the model is able to accurately simulate the interaction of the material and the chemical properties, a reasonable characterization of the asphalt binder and rejuvenator materials and the nature of the material, so as to provide a reliable basis for the study of the asphalt binder blending behavior later on.

#### 1.4.2 Verification of glass transition temperature.

The critical temperature when the asphalt binder changes from viscoelastic to brittle is called the glass transition temperature (Tg), which is usually used to evaluate the low-temperature cracking resistance of asphalt binder materials. To further verify whether the constructed asphalt binder model is reasonable, the glass transition temperature of the asphalt binder model is calculated. According to the thermodynamic theory, the relationship between material specific volume and temperature satisfies different linear relationships in different temperature regions. Therefore, by calculating the density of the asphalt binder model at multiple temperature points, the relation between specific volume and temperature was derived. According to the definition of glass transition temperature, the two parts with different slopes are fitted linearly and the temperature corresponding to the intersection of the resulting two fitting lines is the glass transition temperature of the asphalt binder model [38].

**Fig 10:**
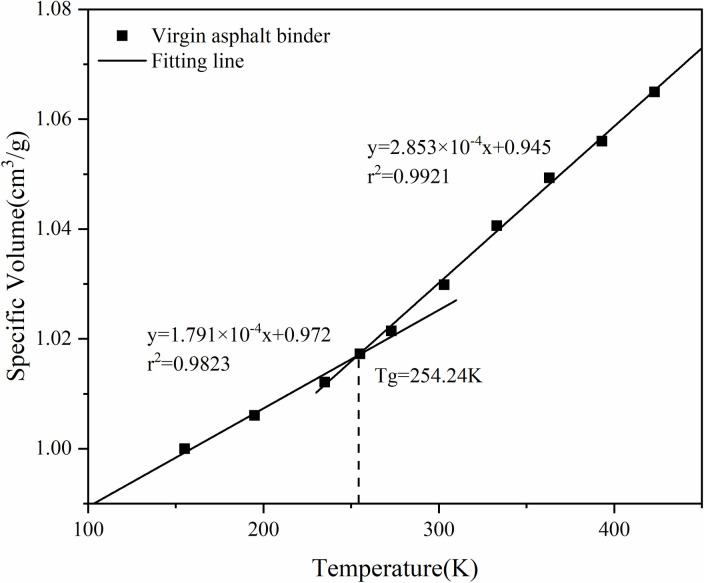
Specific volume versus temperature for virgin asphalt binder models.

**Fig 11:**
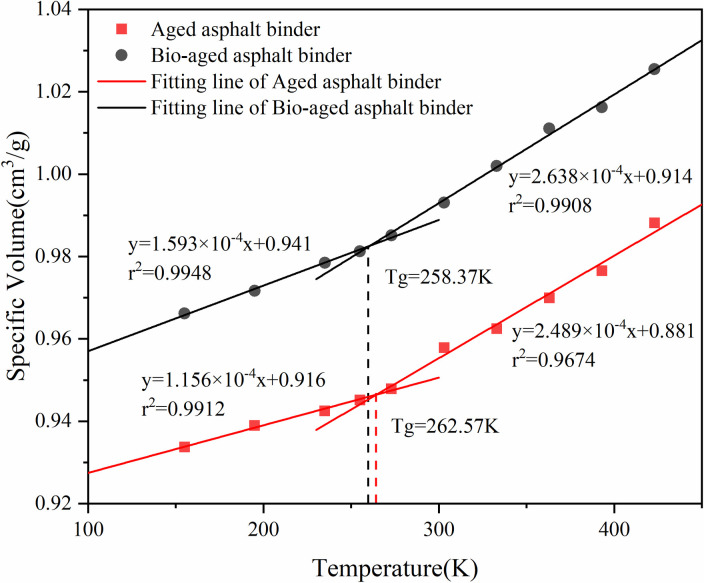
Specific volume versus temperature for aged asphalt binder models.

Ten temperature points, 155K, 195K, 235K, 255K, 275K, 305K, 335K, 365K, 395K, and 425K, were selected to perform molecular dynamics simulations under the NPT system, with a pressure of 0.1 MPa, and to calculate the average density of the asphalt binder after equilibrium at different temperatures. The fitted specific volume-temperature relationship of the asphalt binder model is shown in Figs 10 and 11.

As shown in Figs 10 and 11, two fitted lines are derived from the linear fitting in the high temperature region and the low temperature region respectively, and the temperature corresponding to its intersection point is the glass transition temperature of the asphalt binder molecular model. That is, the glass transition temperature of the virgin asphalt binder molecular model is 254.24 K, that of the aged asphalt binder molecular model is 262.57 K, and that of the bio-aged asphalt binder molecular system is 258.37 K. The glass transition temperature of asphalt binder can be measured by the Differential Scanning Calorimetry (DSC) method, and according to the glass transition temperature of asphalt binder by Lin Zhi [39] using the DSC2500 Differential Scanning Calorimetry, the glass transition temperature of virgin asphalt binder was 253.76 K and 265.94 K for aged asphalt binder, which shows that the molecular simulation results of the glass transition temperature of asphalt binder are consistent with the results of the Differential Scanning Calorimetry test.

### 1.5 Layered blending model construction

**Fig 12:**
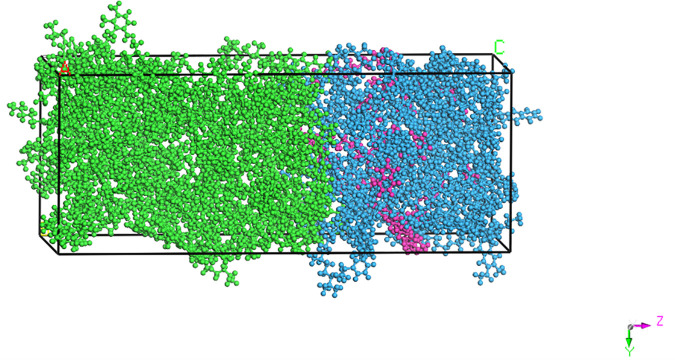
35R15 miscible layer model.

In this study, three kinds of aged asphalt binder content were set, respectively, 25%, 35% and 45%, and three kinds of rejuvenator content are selected, namely 5%, 10% and 15%, and the group without added rejuvenator is set as a comparison. Here the aged asphalt binder content refers to the proportion of aged asphalt binder in the total asphalt binder content of recycled asphalt mixture, combined with past engineering practice, if the total asphalt binder usage is preset to 4.5%, according to the RAP asphalt binder content test results and mix design results to find out the specific content of aged asphalt binder in recycled asphalt mixtures under the aged asphalt binder content of 25%, 35%, 45%, respectively. At 35% content, if the aged asphalt binder content is found to be 1.45%, and the rejuvenator content is 10% of the aged asphalt binder content, then the virgin asphalt addition is 3.05%. For the convenience of representation, the model containing 35% aged asphalt binder content, 10% rejuvenator content is referred to as 35R10 model, and so on. According to the previous method to construct the virgin asphalt binder model and the bio-aged asphalt binder model, the temperature is 433K, and then use the ‘Build Layer’ module for the virgin and aged asphalt binder cell model obtained above to establish the virgin-aged asphalt binder layered blending model shown in Fig 8. To 35R15, for example, according to the previous method for its structure optimization and system relaxation to obtain a stable blending model, the model with dimensions of a = b = 33.06 Å, b = 33.06 Å, b = 33.06 Å, c = 83.46 Å, and the energy is 27893.7 kcal/mol. 35R15 blending model is shown in Fig 12. The virgin asphalt binder molecules were set to green, the aged asphalt binder molecules to blue, and the rejuvenator molecules to pink for ease of observation.

## 2 Results and discussion

### 2.1 Energy analysis of the blending process

The molecular diffusion process is driven by several factors, including molecular thermal motion, van der Waals interactions, and Coulomb interactions. The systems with different contents of aged asphalt and different rejuvenator contents were simulated by blending respectively, and their intermolecular interaction energies are shown in .

**Fig 13:**
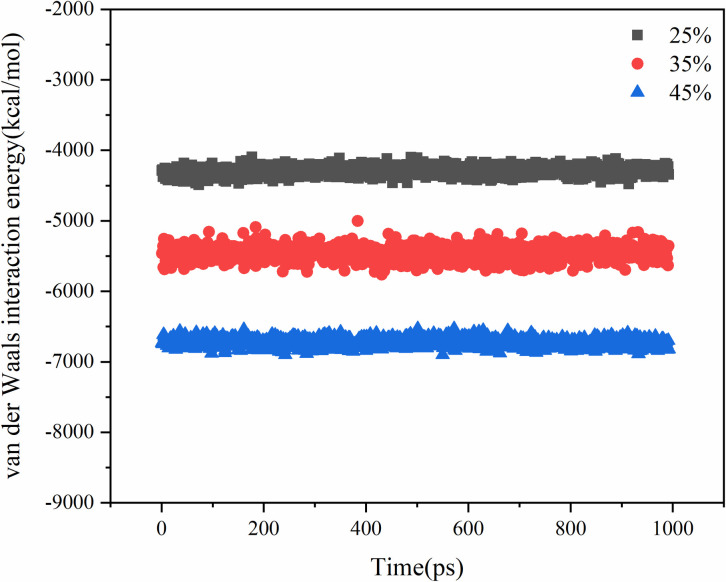
Van der Waals interaction energy analysis of different aged asphalt content.

**Fig 14:**
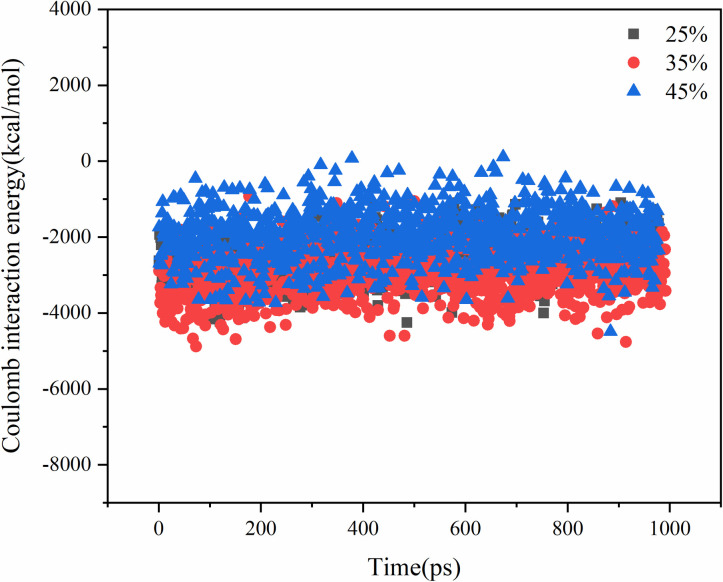
Coulomb interaction energy analysis of different aged asphalt content.

**Fig 15:**
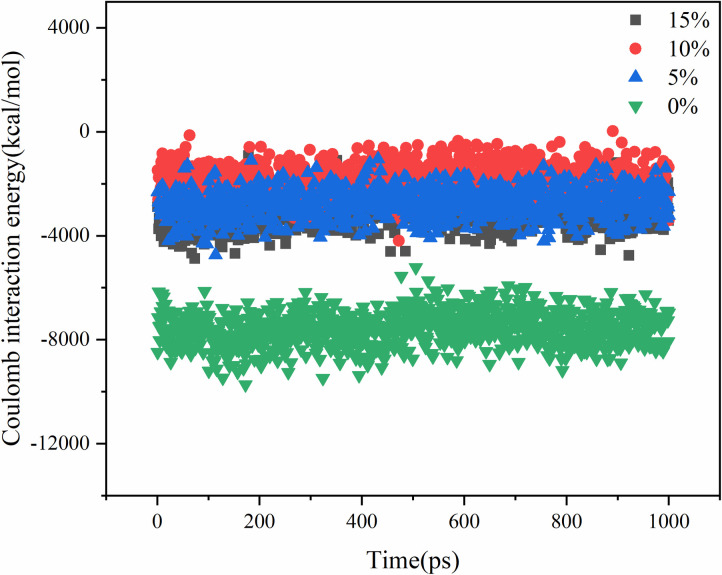
Coulomb interaction energy analysis of different rejuvenator content.

**Fig 16:**
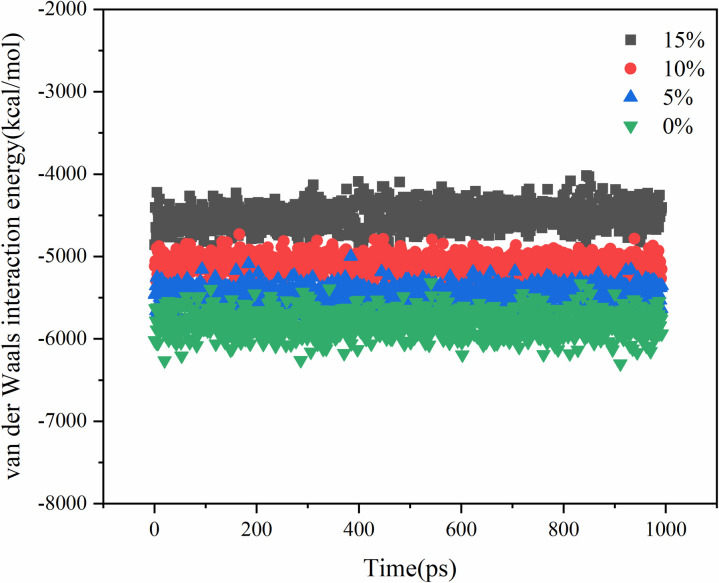
Van der Waals interaction energy analysis of different rejuvenator content.

The Coulomb interaction energy was found to be not very responsive to the change in the content of virgin and aged asphalt, and the difference between the Coulomb interaction energies of the different virgin and aged asphalt models was within 5%. The van der Waals interaction energy values, increased with the increase in the content of aged asphalt. Therefore, the Van der Waals forces may play a major role in the mutual diffusion of virgin and aged asphalt. On the other hand, from the aspect of asphalt structure, asphalt is a system composed of several molecular clusters with different polarities, which relate to each other by non-polar lightweight components. Some of the lightweight components are lost when aged asphalt, which increases the attraction between virgin and aged asphalt when they are blended due to the difference in polarity. That is, the addition of rejuvenator molecules to change the original polar molecular interactions in the system, resulting in a decrease in the overall interaction energy, but at the same time improve the molecular activity, thus promoting the process of blending.

### 2.2 Calculation of the diffusion coefficient

In this study, the diffusion coefficient calculated on the basis of Mean Square Displacement (MSD) was used as a key parameter for evaluating the degree of blending.The slope of the MSD variation curve is closely related to the diffusion coefficient, and 1/6 of the slope can be regarded as the diffusion coefficient, which reflects the rate of diffusion of molecules in the system with the passage of time.

The mean square displacement (MSD) of particle motion is defined as the fact that liquid and gas molecules do not remain fixed in one position; their positions are constantly changing. Its definition is shown in equation (3).


$$MSD(t)=\left\langle {\left|r_{i}(t)-r_{i}(0)\right|}^{2}\right\rangle \mathrm{\backslash ]}$$ (3)

Where:

ri (t)--Particle i’s position vector at moment t;

ri (0) – the position vector of particle i at the initial moment;

<> - averaged over the entire simulation time for all particles in the system.

The limiting slope of the MSD (mean square displacement) is a function of time and can be used to evaluate the diffusion coefficient D for random Brownian motion of particles in three-dimensional space as shown in Equation (4).


$$D=\frac{1}{6}\lim_{t\to \infty }\frac{d\left(MSD(t)\right)}{dt}\approx \frac{k}{6}\mathrm{\backslash ]}$$ (4)

Where, K-slope of the linear correlation section between mean square displacement and time.

**Fig 17:**
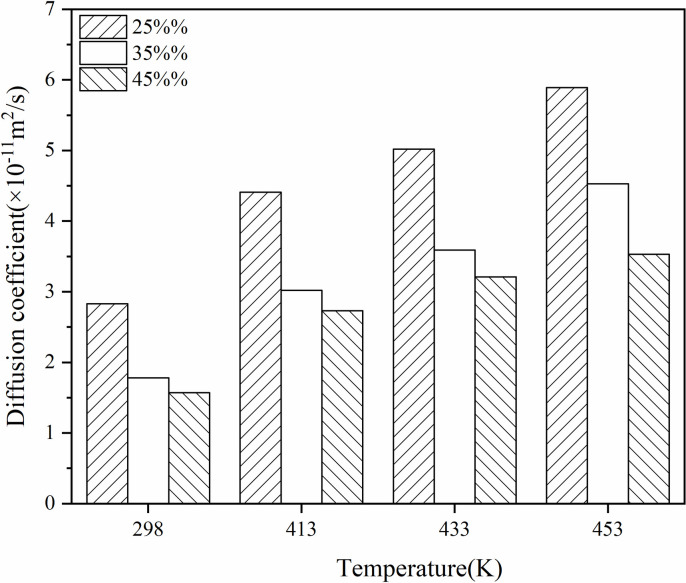
Diffusion coefficients of aged asphalt in models with different aged asphalt content.

**Fig 18:**
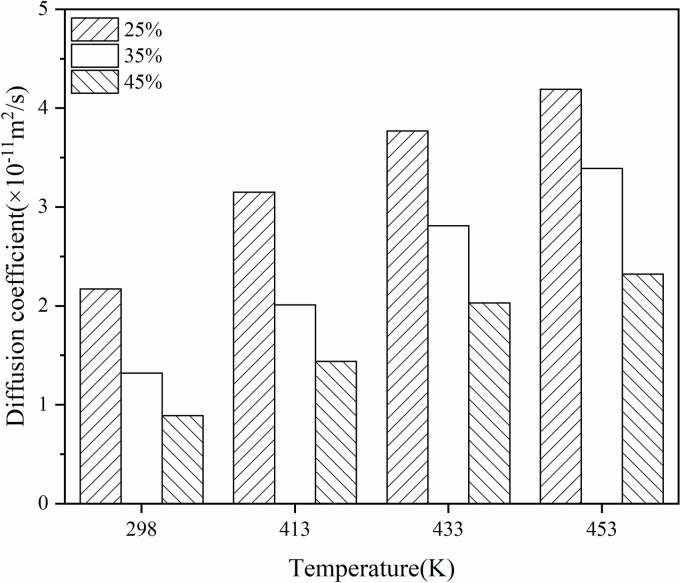
Diffusion coefficients of virgin asphalt in models with different aged asphalt content.

Derive the distribution of the mean square displacement of virgin and aged asphalt molecules in the system with different contents of aged asphalt and analyse the fitting of MSD. Calculate the diffusion coefficients of rejuvenator and asphalt based on the fitting results. As shown in Figs 17 and 18, the reported figures are derived from a single simulation.

Temperature was found to promote blending, and the increase in the content of virgin and aged asphalt slowed down the process of mutual blending of virgin and aged asphalt and had a greater effect on the diffusion of the old asphalt. At the same time, when the content of aged asphalt increased from 25% to 35%, the diffusion coefficient decreased significantly more than when the content increased from 35% to 45%. This indicates that the diffusion coefficient starts to decrease significantly after the aged asphalt content exceeds 35%, so the old asphalt content should not be higher than 35% in terms of diffusion coefficient considering the actual engineering efficiency.

### 2.3 Calculation of the degree of blending

The blending model of virgin and aged asphalt binder will begin to diffuse into each other at the interface between the virgin and aged asphalt binder, the model of blended asphalt binder after mutual diffusion can be divided into three parts, respectively, for the virgin asphalt binder region, the aged asphalt binder region, and the middle part of the virgin and aged asphalt binder blended region. With the growth of the simulation time, the scope of the blended region will gradually increase, however, since there is a difference between the virgin and aged asphalt binder solubility parameters, the virgin and aged asphalt binder molecules are difficult to tightly align to form a new phase, it is difficult to achieve complete blend.

In this study, DOB (degree of blending) was used to quantitatively characterize the blending state of virgin and aged asphalt binder in recycled asphalt binder, and the degree of blending of virgin and aged asphalt binder was calculated by calculating the percentage of the mass of aged asphalt binder located in the blended region in relation to the total mass of aged asphalt binder [18], as shown in Eq.(5):


$$DOB=\frac{m}{M}\times 100\%\mathrm{\backslash ]}$$ (5)

Where m is the mass of aged asphalt binder that was blended with the virgin asphalt binder; M denotes the total mass of aged asphalt binder in the model.

Degree of Blending (DOB) is typically quantified using metrics such as mass distribution, but its core reflects spatial overlap (physical blending). If the molecules of aged and virgin asphalt achieve spatially uniform distribution (e.g., no significant phase separation) within the simulation time, the DOB value is high, indicating good physical blending.

However, a key limitation is that DOB only reflects statistical overlap of molecular positions and cannot directly confirm the presence of chemical bonding or thermodynamic compatibility. To enhance its predictive reliability, multi-scale methods, experimental validation, and force field optimization are required.

**Fig 19:**
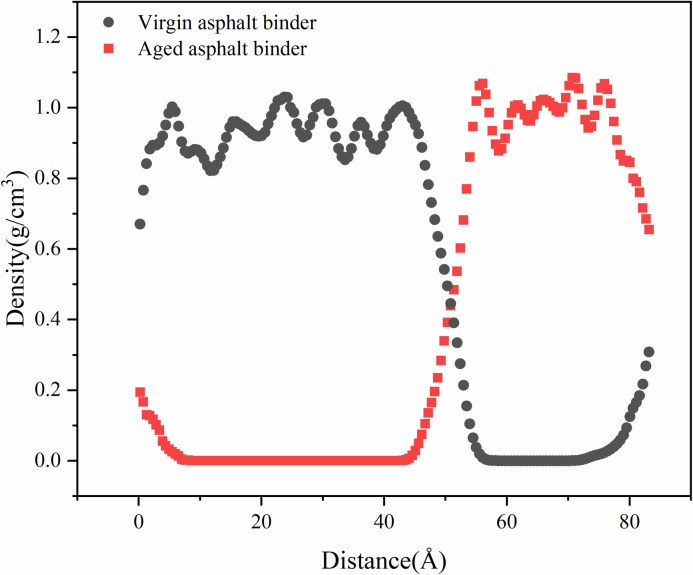
Mass density distribution of recycled asphalt binder models.

The range of blended region was determined based on the mass-density distribution, which is shown in Fig 19. Take 35% aged asphalt binder content model as an example, the range of virgin asphalt binder is 0–59 Å, the range of aged asphalt binder is 41–83 Å, then the blended region range is 41–59 Å. The molecular mass of the aged asphalt binder located in the blended region is counted and its ratio to the total mass of the aged asphalt binder is calculated to obtain the degree of blending.

### 2.4 Analysis of the degree of blending

**Fig 20:**
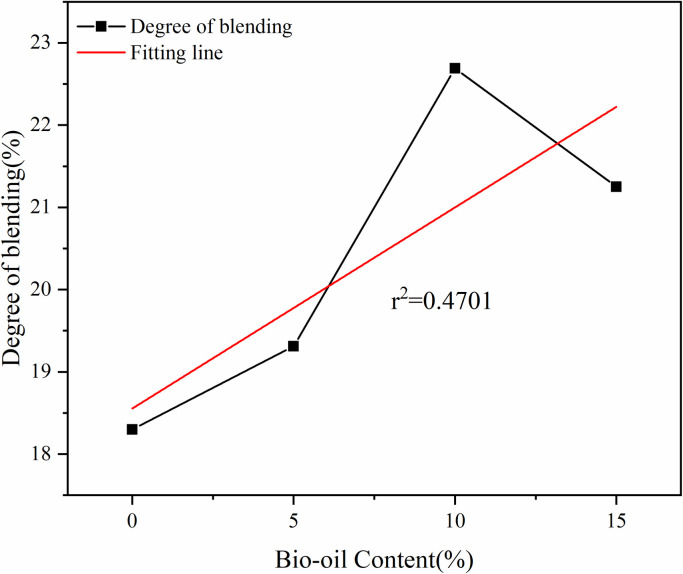
Effect of rejuvenator content on degree of blending.

**Fig 21:**
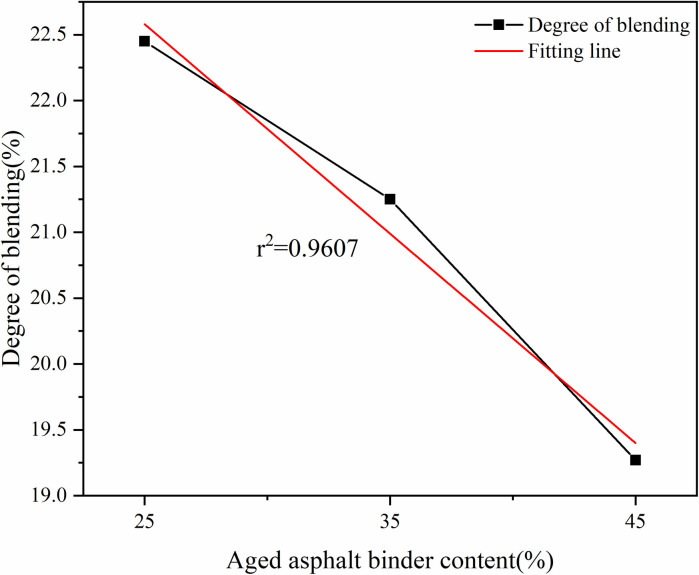
Effect of aged asphalt binder content on degree of blending.

**Fig 22:**
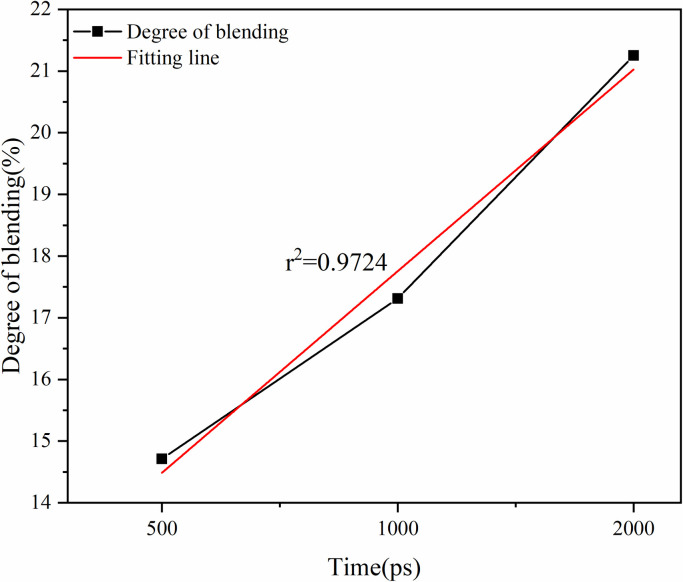
Effect of simulation time on degree of blending.

**Fig 23:**
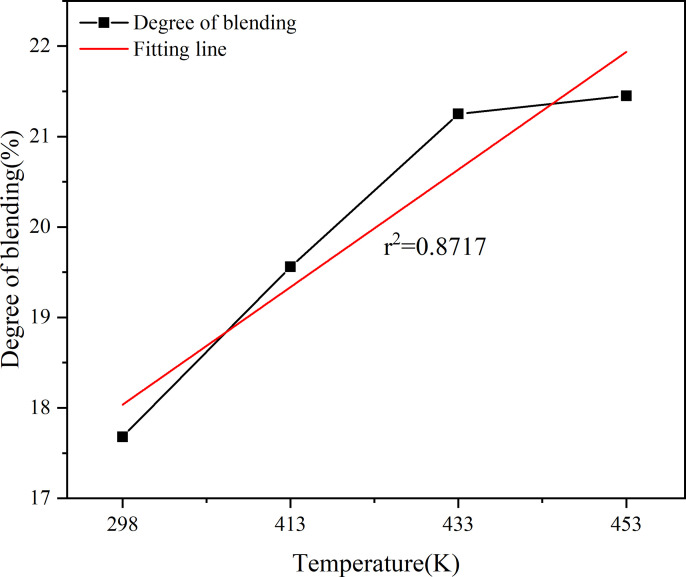
Effect of simulation temperature on degree of blending.

The changes in the degree of blending were analyzed from four aspects: rejuvenator content, aged asphalt binder content, temperature and simulation time. When analyzing the effect of rejuvenator content, 35% of aged asphalt binder content is selected and the temperature is 433K; when analyzing the effect of aged asphalt binder, 15% of rejuvenator content is selected and the temperature is 433K; and when analyzing the effect of temperature and simulation time, all of them are based on the model of 35R15 as an example. The changes in the degree of blending are shown in Fig 10(a), 10(b), 10(c) and 10(d). To further determine the effects of the four factors on the degree of blending, the four factors were fitted separately, and the fitted lines are shown as the red lines in Figs 20–23, the reported figures are derived from a single simulation.

From Fig 20, with the increase in the content of rejuvenator, the degree of blending shows a gradual growth trend, but to 15% have decreased, which may be due to the increase in the number of model molecules. The increase in the degree of blending is more significant when the rejuvenator content is increased from 5% to 10%.

On one hand, the increase in small-molecule substances in the rejuvenator improves the fluidity of the system, acting as a lubricant. On the other hand, it weakens the interactions between polar molecules, promoting the uniform distribution of aged asphalt. These effects lead to a reduction in the cohesive energy density of the asphalt system, thereby increasing the diffusion coefficient. However, the rate of increase gradually diminishes with higher dosages. This phenomenon may be attributed to the fact that asphaltenes in asphalt often exhibit π-π stacking of polycyclic aromatic hydrocarbons. The polycyclic aromatic structures attract each other, and aging introduces more double bonds in the asphalt, which to some extent enhances the mutual attraction between asphaltenes. As a result, molecules in aged asphalt are more likely to stack, with shorter stacking distances. After adding the rejuvenator, the aromatic molecules in the rejuvenator interact with the asphaltenes through aromatic ring stacking, causing the asphaltene molecules to move or distort. Meanwhile, chain-like carbon-oxygen compounds or aliphatic hydrocarbons can disrupt the interactions between the polycyclic aromatic structures of asphaltenes. Both types of molecules can effectively separate asphaltenes and disrupt their stacking.

It can be seen from Fig 21 that the degree of blending shows a gradual decrease with the increase in the content of the aged asphalt binder. After fitting, it was found that the r^2^ value of rejuvenator content was 0.4701, and the r^2^ value of aged asphalt binder content was 0.9607.

As shown in Fig 22, the degree of blending of the virgin and aged asphalt binder increases with the growth of the simulation time, but the virgin and aged asphalt binder still do not reach complete blend after diffusion for 2 ns, and the virgin and aged asphalt binder here are only preliminary blend. As shown in Fig 23, the degree of blending increases with the temperature, but the growth is slowed down after going from 433 K to 453 K. The r^2^ value of the simulation time is 0.9724, and the r^2^ value of the temperature is 0.8717. After fitting, it is found that the r^2^ value of simulation time is 0.9724, and the r^2^ value of temperature is 0.8717.

Based on the fitting results, the correlation between the four factors and the degree of blending is in the following order: simulation time > aged asphalt content > temperature > rejuvenator content, where the old asphalt content is negatively correlated, and the rest are positively correlated.

## 3 Experimental validation conclusions

### 3.1 DSR test validation

In this study, the macroscopic blend behavior of virgin and aged asphalt blend systems was experimentally investigated using a dynamic shear rheometer (DSR). The test was carried out using the DHR-1 rheometer produced by the European and American Earth Science and Technology Group. The dynamic shear rheology test was carried out in temperature scanning mode with the following parameters: temperature range 46–82°C, loading frequency 10 rad/s and strain level 12%. A 25 mm diameter plate was used with a test gap of 1 mm and a temperature increase rate of 2 °C/min.

In the test, the blending temperatures were set at 120°C, 140°C and 160°C, and the blending times were 20 min, 40 min, 60 min and 80 min, and the recycled asphalt specimens were named according to the order of ‘test indexes - blending conditions’, e.g., ‘G*/sin δ-20min’ means the G*/sin δ value measured after 20 minutes of blending of the old and new asphalt. For example, ‘G*/sinδ-20min’ means the G*/sin δ value measured after 20 minutes of blending virgin and aged asphalt. The rutting factor and phase angle were fitted according to polynomial and linear, and the results are shown in .

**Fig 24:**
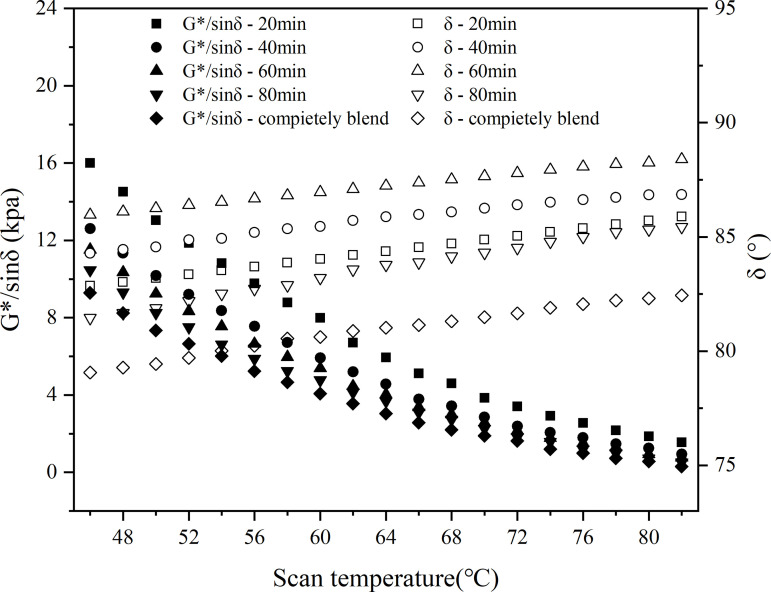
Rutting factor and phase angle at 120°C.

**Fig 25:**
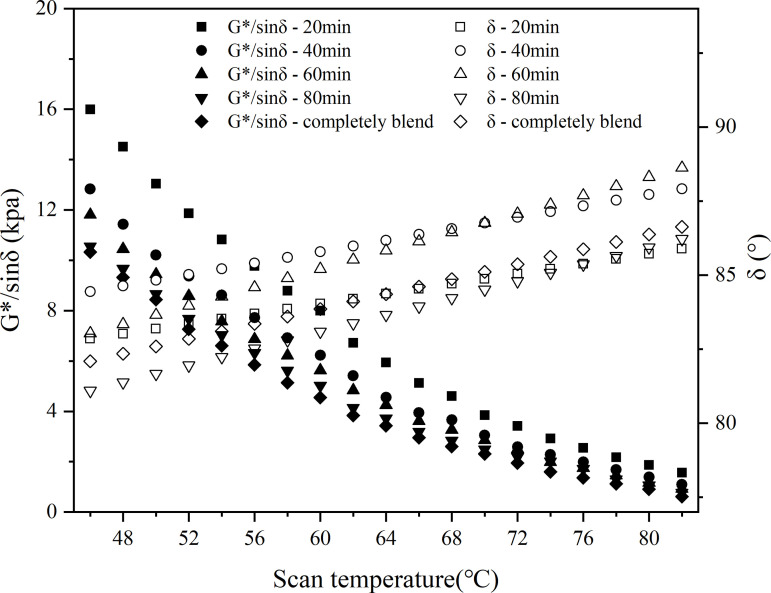
Rutting factor and phase angle at 140°C.

**Fig 26:**
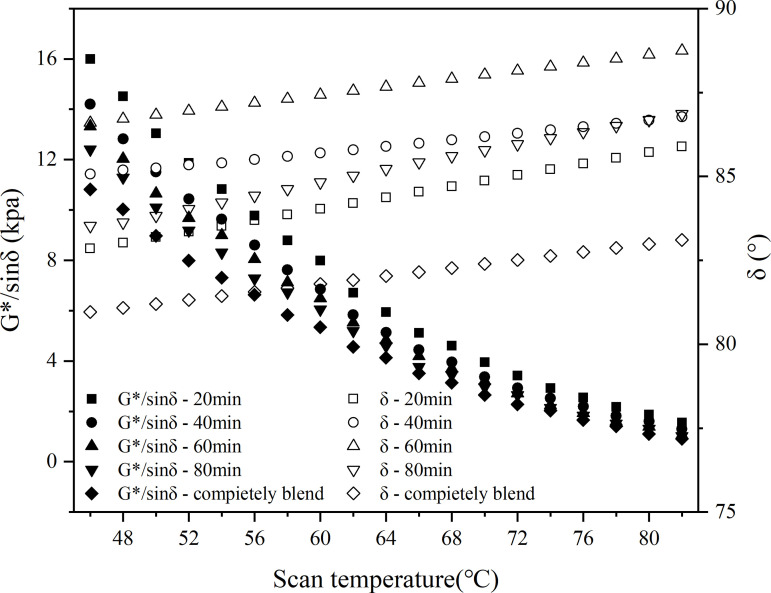
Rutting factor and phase angle at 160°C.

The temperature scanning curves showed that the G*/sinδ values of the samples with different blending times were in the following order: 20min < 40min < 60min < 80min, indicating that the longer the blending time, the higher the G*/sinδ value, and this law existed at different blending temperatures; meanwhile, at different blending temperatures, the G*/sinδ values were in the following order from small to large: 120°C < 140°C < 160°C. The higher the temperature, the higher the G*/sinδ value, the higher the G*/sinδ value. 140°C < 160°C, and the higher the temperature, the larger the G*/sinδ value. From the change of phase angle, the phase angle of partially blended asphalt was basically located between 78° and 90°, and the phase angle of blended asphalt was roughly in the order of blending time from largest to smallest as 40min > 20min>completely blended>60min > 80min.

### 3.2 Experimental validation of three major indicators

Firstly, 10% content rejuvenator was blended with aged asphalt, held at 140°C oven for 30 min, and sheared at 3000r/min for 30 min to prepare rejuvenated asphalt. Then the virgin asphalt and 25%, 35%, 45% dosage of recycled asphalt blend, respectively, placed in 120°C, 140°C, 160°C conditions of heat preservation for 80 min, the use of three major indicators to evaluate the impact of aged asphalt dosage on recycled asphalt.

**Fig 27:**
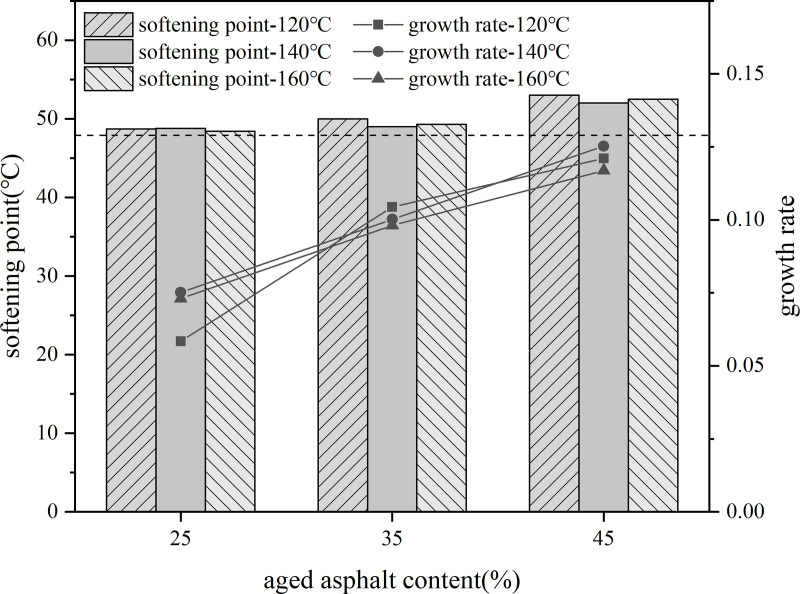
Softening point and rate of change of different aged asphalt content.

**Fig 28:**
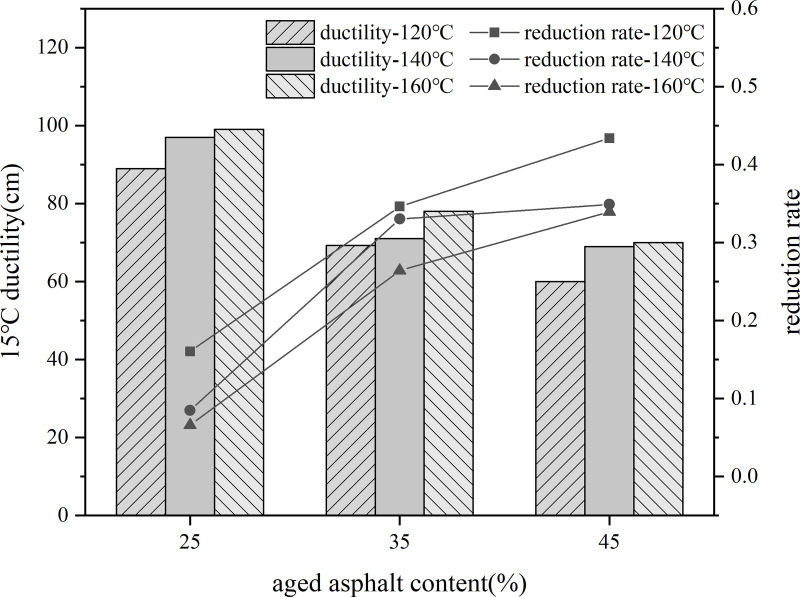
15°Cductility and rate of change of different aged asphalt content.

**Fig 29:**
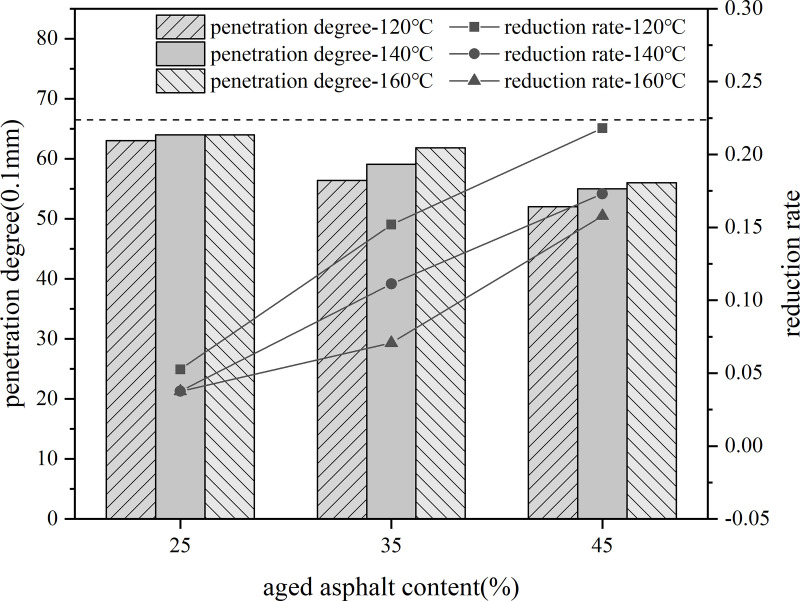
Penetration degree and rate of change of different aged asphalt content.

Using different aged asphalt content of recycled asphalt model indicator value based on the rate of change of the indicator value of virgin asphalt as the vertical coordinate of the line graph. As shown in the Figs 27–29.

As the content of aged asphalt increases, the difference between the index values of needle penetration, softening point and ductility of recycled asphalt and the index value of virgin and aged asphalt is larger, indicating that the increase in the amount of old asphalt will lead to the weakening of the degree of blending of old and new asphalt.

5%, 10%, 15% content of rejuvenator were added to the aged asphalt, and in the 140°C-oven insulation penetration for 30 min, 3000r/min speed shear 30 min to prepare recycled asphalt. Three kinds of rejuvenated asphalt with no added rejuvenator aged asphalt at 35% content were blended with virgin asphalt, and were placed in the 120°C, 140°C, 160°C conditions of heat preservation for 80 min, the use of the three major indicators and their rate of change in the evaluation of rejuvenation agent dosage of rejuvenated asphalt.

**Fig 30:**
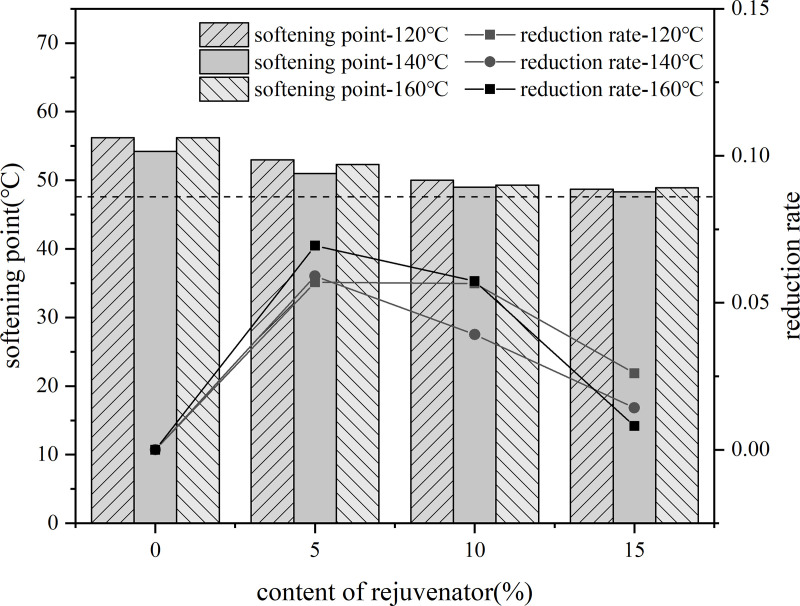
Softening point and rate of change of different rejuvenator contents.

**Fig 31:**
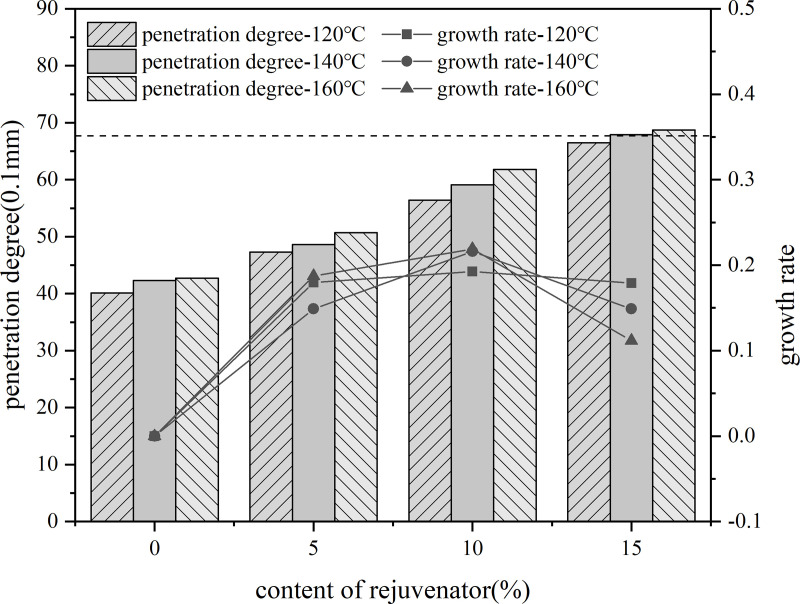
Penetration degree and rate of change of different rejuvenator contents.

**Fig 32:**
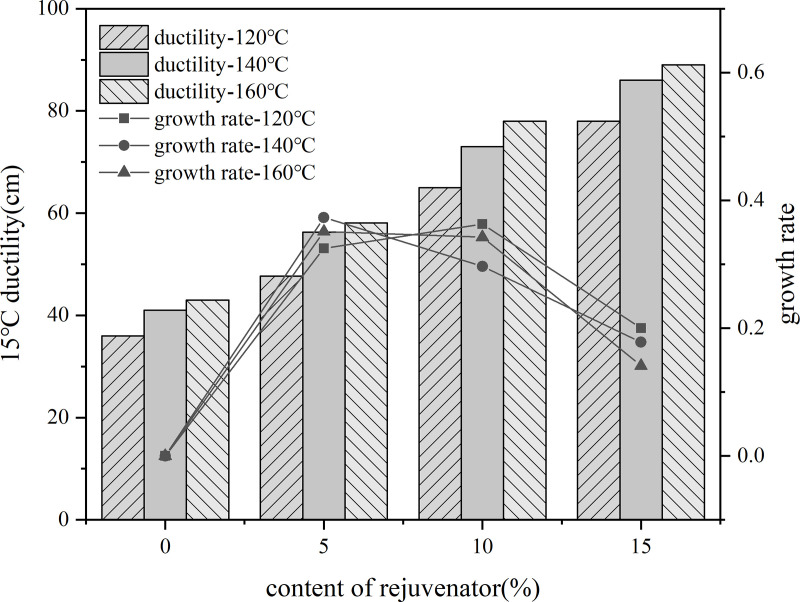
15°Cductility and rate of change of different rejuvenator contents.

A line graph was plotted using the rate of change of the indicator values of the current rejuvenator content asphalt model based on the rate of change of the indicator values of the previous rejuvenator content asphalt model as the vertical coordinate. As shown in the Figs 30–32.

The results show that, with the increase of rejuvenator content, the performance indicators of reclaimed asphalt gradually converge to the virgin asphalt index value, but its rate of change shows a decreasing trend. When the content reached 10%, the rate of change of indicators show a decreasing trend. Therefore, the selection of bio-oil rejuvenator BR when the content should be controlled at 15% or less. When the dosage increased to 10%, the needle penetration and softening point just to meet the performance requirements, so the dosage of bio-oil rejuvenator is recommended to choose 10% to 15% or so.

As shown in Section 2.4, the simulation time and temperature for the blending of aged and virgin asphalt are positively correlated with the blending degree. The r² value for simulation time is 0.9724, while that for temperature is 0.8717. The r² values for rejuvenator content and aged asphalt content are 0.4701 and 0.9607, respectively.

The ratio of the rutting factor between partially blended and fully blended asphalt was used as an evaluation index for the blending degree of aged and virgin asphalt, denoted as $P_{G^{*}/\sin\delta }$, as shown in Eq. (6):


$$P_{G^{*}/\sin\delta }=\frac{G_{t_{1}}^{*}/\sin\delta_{t_{1}}}{G_{t_{0}}^{*}/\sin\delta_{t_{0}}}\mathrm{\backslash ]}$$ (6)

where:

$P_{G^{*}/\sin\delta }\backslash )$ represents the evaluation index for the blending degree of aged and virgin asphalt, (%);

$G_{t_{1}}^{*}\backslash )$ and $G_{t_{0}}^{*}\backslash )$ denote the complex modulus of partially blended and fully blended asphalt specimens, (kPa);

$\delta_{t_{1}}\backslash )$ and $\delta_{t_{0}}\backslash )$ represent the phase angles of partially blended and fully blended asphalt at temperature t °C, (°).

Four scanning temperatures—52°C, 58°C, 64°C, and 70°C—were selected to calculate the blending degree evaluation index. A correlation analysis was conducted between the blending temperature and blending time obtained from rheological tests and the blending degree evaluation index P, with the results averaged. Additionally, the changes in conventional performance indicators (penetration, softening point, and ductility) were analyzed through fitting.

The average r² value for the correlation between blending time and the blending degree evaluation index was 0.9667, while that for blending temperature was 0.8555. The average r² values for the conventional performance indicators showed that the correlation between aged asphalt content and fundamental performance indicators was 0.9493, whereas that for rejuvenator content was only 0.2215.

By integrating the results from rheological tests and conventional performance tests, the influence of the four factors on the blending degree was ranked as follows: blending time > aged asphalt content > blending temperature > rejuvenator content. This ranking aligns with the molecular dynamics simulation results presented earlier, confirming the accuracy of the molecular dynamics analysis in studying the blending behavior of aged and virgin asphalt in recycled asphalt. These findings provide valuable guidance for the preparation and performance optimization of recycled asphalt mixtures in engineering applications.

## 4 Conclusions and limitions

In this study, a four-component 12-molecule molecular model of virgin and aged asphalt binder was established by molecular dynamics simulation, a representative molecular model of bio-oil rejuvenator was selected, and a layered blending model was assembled by using Materials Studio software in accordance with different content, to investigate the blending behavior of the virgin and aged asphalt binder in the hot in-plant recycled asphalt mixture. The effects of temperature, rejuvenator content, aged asphalt binder content and simulation time on the degree of blending of virgin and aged asphalt binder were investigated, and the following conclusions were obtained:

(1) Rejuvenator content of 10% or less, the degree of blending with the increase in the content of rejuvenator showed a gradual increase in the trend, to 15% decreased, indicating that the appropriate addition of rejuvenator is conducive to the blending of the virgin and aged asphalt binder, but it is not the more the better. The fitted r^2^ value is 0.4701, which shows that there is no obvious linear relationship between the content of rejuvenator and the degree of blending.
(2) The higher the content of aged asphalt binder, the lower the degree of blending. The r^2^ value of 0.9607 can be seen from the fitted line in the figure, indicating that there is a strong negative correlation between the aged asphalt binder content and the degree of blending, which shows an obvious linear relationship.
(3) With the growth of simulation time, the degree of blending of virgin and aged asphalt binder will continue to increase, but due to time constraints, this study did not carry out longer blending simulation. The fitted r^2^ value is 0.9724, which shows that there is a strong positive correlation between the degree of blending and the simulation time within 2000ps.
(4) The degree of blending of the virgin and aged asphalt binder increased with increasing temperature. The increasing trend of degree of blending is more significant when the temperature increases from 298K to 433K, in which the degree of blending increases by 13.7% from 298K to 413K, and by 10.8% from 413K to 433K, while the degree of blending only increases by 1.2% when the temperature increases from 433K to 453K.
(5) According to the fitting results, the correlation between the four factors and the degree of blending is in the following order: simulation time > aged asphalt binder content > simulation temperature > rejuvenator content, in which the aged asphalt binder content is negatively correlated and the rest is positively correlated.
(6) Comparative analysis of the molecular dynamics simulation results and the macroscopic test results shows that there is a good correlation between the parameters at the molecular level and the macroscopic properties of the asphalt binder, which also indicates the feasibility of molecular simulation for the study of asphalt materials and the reasonableness of the results.
(7) Due to the test conditions and time constraints, the changes in the four components of aged asphalt and their effects on the blend were not analyzed in depth. This direction can be followed to carry out in-depth research.

## Supporting information

S1 Data Original dataset. (RAR)
